# 4DCT Differentiation of Parathyroid Adenoma: A Case Report

**DOI:** 10.5334/jbsr.3238

**Published:** 2023-08-17

**Authors:** Tana Bupe Mwewa, Steven Raeymaeckers

**Affiliations:** 1 UZ Brussel, Belgium

**Keywords:** CT, Head and Neck, Hyperparathyroidism, Parathyroid Adenoma

## Abstract

We report the case of a 72-year-old patient presenting with recurrent primary hyperparathyroidism after total thyroidectomy and parathyroidectomy with presternal autotransplantation. Methionine-PET-CT proved false-positive and Tc-99m-tetrofosmine imaging false-negative. Using a novel multiphase-4DCT technique we identified an anterior mediastinal nodule demonstrating contrast wash-in and wash-out, suggesting parathyroid adenoma. Traditional 4DCT-protocols obtain fewer phases; therefore, this enhancement pattern might have gone unnoticed. After surgical resection the bloodwork normalized, histopathology confirmed a parathyroid adenoma.

**Teaching Point:**

Multiphase 4DCT is a potentially helpful technique for the detection of parathyroid adenomas after total thyroidectomy and parathyroidectomy.

## Introduction

Primary hyperparathyroidism is a common endocrine disease. Conservative treatment is possible in asymptomatic patients over the age of 50 without end-organ complications, but the only cure is surgery [1,2]. Bilateral neck exploration is being abandoned as treatment standard in favour of minimally invasive surgery, aided by rapid parathyroid hormone determination techniques and effective preoperative imaging [3]. Four-dimensional computed tomography (4DCT) is commonly performed to localize enlarged parathyroid glands, combining three-dimensional imaging with contrast enhancement over time as the fourth dimension. 4DCT scanning protocols for parathyroid adenoma detection are institution-specific, typically obtaining three or four contrast phases [4]. Our hospital adopted a novel scanning technique consisting of 16 contrast phases, as described by Raeymaeckers et al. in 2021 [5], which can provide more detailed enhancement patterns and allow for better tissue classification, as this case demonstrates.

## Case History

A 72-year-old patient with a history of total thyroidectomy and parathyroidectomy with presternal autotransplantation two years prior, presented with recurrent primary hyperparathyroidism (iPTH 174ng/l; calcium 2.56 mmol/l) while under treatment with Mimpara. Hyperplasia of the presternal autotransplant was considered, but an older Methionine-PET-CT from another hospital seemingly indicated a hotspot in the upper mediastinum, to the right of the trachea. To confirm a supernumerary mediastinal parathyroid, Tc-99m-tetrofosmine scintigraphy was performed, which failed to show ectopic captation. Surgeons were reluctant to operate without more certainty on the localization, wishing to avoid a bilateral exploration in this postoperative patient. Multiphase-4DCT was performed, demonstrating a parathyroid adenoma in the left base of the neck, between the common carotid artery (CCA) and subclavian vein. This supernumerary parathyroid was surgically resected. Postoperative bloodwork showed normalized blood calcium (2.18 mmol/l) and iPTH (16.8 ng/l). Histopathology confirmed parathyroid adenoma in the resected tissue.

## Discussion

4DCT allows the evaluation of enhancement patterns over time and can detect abnormal parathyroid glands with a sensitivity of 85.7% [6]. Bahl et al. [7] considered three enhancement patterns: all parathyroid adenomas appear hypoattenuating to thyroid tissue on non-contrast-enhanced imaging (NECT). Lesions may show avid arterial hyperenhancement (type A, 20%); rapid venous phase wash-out (type B, 57%); or neither (type C, 22%). 4DCT is considered useful for the detection of ectopic glands or in case of persistence/recurrence after surgery [8]. Standard 4DCT-protocols typically include three/four phases; arterial images are usually obtained 25–30 seconds after contrast administration, the timing of the venous phase(s) varies greatly in literature [4].

In our hospital, we obtain 16 phases. Scanning happens on a 256-slice Revolution CT (GE Healthcare). A venous catheter is placed in the patient’s cubital vein, their arms in a neutral position alongside the body, head fixed in a head cradle. The sensation of contrast administration is explained, and the patient is instructed not to move or swallow. Scanning is centred on the thyroid over a fixed 16 cm coverage volume (100 kVp, SmartmA 10–480 mA, thickness 0.625 mm, 0.5 s rotation scanning time). Wide-beam axial scanning is chosen over helical to limit dosage. This protocol has a mean effective dose of 6.7 mSv (can be as low as 1.4 mSv), whereas the effective dose of 4DCT-protocols in literature is 10.4 to 13.8 mSv [5].

**Figure 1:**
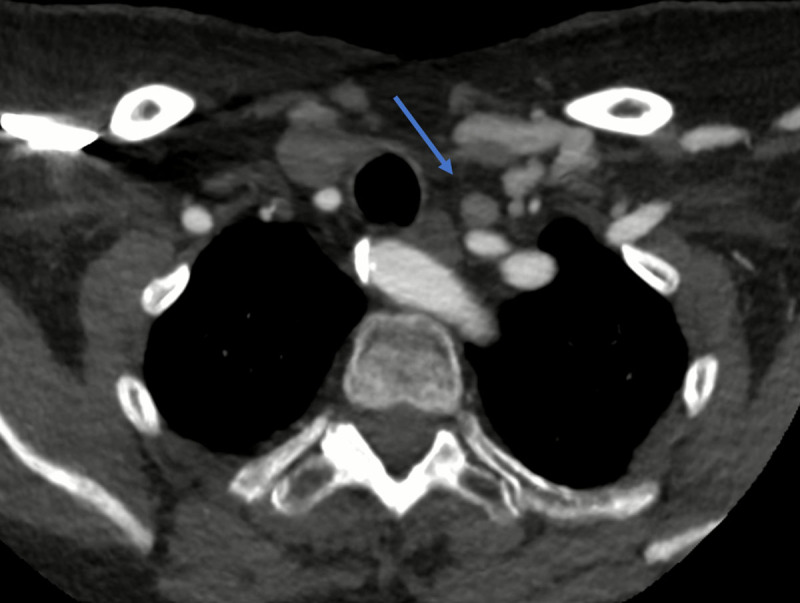
Axial arterial-phase image of the nodule anterior to the left CCA.

**Figure 2:**
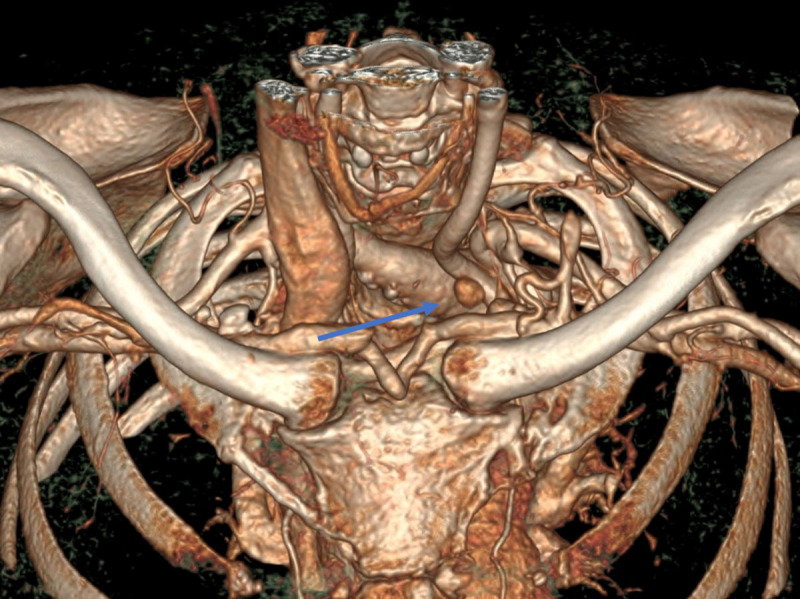
3D-reconstruction showing the anterior mediastinal nodule.

**Figure 3:**
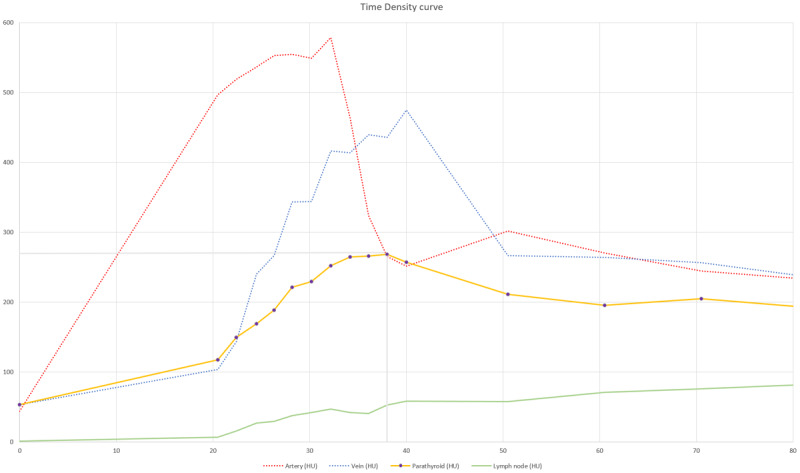
Time-density curve; note wash-in and wash-out.

In this case an 11 mm nodule was found anterior to the left CCA (Figures 1, 2). NECT density is 50 HU – comparison to thyroid is impossible post-thyroidectomy. After contrast administration, reconstructed time-density curves demonstrated both wash-in and wash-out (Figure 3). The maximal density (HUmax) of the structure is 280 HU, much higher than lymphoid tissue. Time to peak (TTP) is 38 seconds, significantly later than most 4DCT-protocols obtain arterial images. This, in combination with the ectopic localisation, could have led to a misclassification as lymphoid tissue without considering the enhancement pattern on the reconstructed time-density curves.

## Conclusion

A 72-year-old patient presented with recurrent primary hyperparathyroidism after total thyroidectomy and parathyroidectomy with presternal autotransplantation. Methionine-PET-CT proved false-positive and Tc-99m-tetrofosmine scintigraphy false-negative. Novel multiphase-4DCT showed an anterior mediastinal nodule demonstrating contrast wash-in and wash-out, suggesting parathyroid adenoma. After surgical resection, bloodwork normalized and histopathology confirmed diagnosis.

## References

[1] Bilezikian JP, Brandi ML, Eastell R, et al. Guidelines for the management of asymptomatic primary hyperparathyroidism: Summary statement from the Fourth International Workshop. J Clin Endocrinol Metab. 2014; 99(10): 3561–9. DOI: 10.1210/jc.2014-1413 25162665 PMC5393490

[2] Silverberg SJ, Clarke BL, Peacock M, et al. Current issues in the presentation of asymptomatic primary hyperparathyroidism: Proceedings of the Fourth International Workshop. J Clin Endocrinol Metab. 2014; 99(10): 3580–94. DOI: 10.1210/jc.2014-1415 25162667 PMC5393491

[3] Udelsman R, Åkerström G, Biagini C, et al. The surgical management of asymptomatic primary hyperparathyroidism: Proceedings of the Fourth International Workshop. J Clin Endocrinol Metab. 2014; 99(10): 3595–606. DOI: 10.1210/jc.2014-2000 25162669

[4] Raeymaeckers S, Tosi M, de Mey J. 4DCT scanning technique for primary hyperparathyroidism: A scoping review. Radiol Res Pract. 2021; 2021: 6614406. DOI: 10.1155/2021/6614406 34094599 PMC8163538

[5] Raeymaeckers S, De Brucker Y, Vanderhasselt T, Buls N, de Mey J. Detection of parathyroid adenomas with multiphase 4DCT: Towards a true four-dimensional technique. BMC Med Imaging. 2021; 21(1): 64. DOI: 10.1186/s12880-021-00597-1 33827463 PMC8028189

[6] Starker LF, Mahajan A, Björklund P, et al. 4D parathyroid CT as the initial localization study for patients with de novo primary hyperparathyroidism. Ann Surg Oncol. 2011; 18(6): 1723–8. DOI: 10.1245/s10434-010-1507-0 21184187

[7] Bahl M, Sepahdari AR, Sosa JA, Hoang JK. Parathyroid adenomas and hyperplasia on four-dimensional CT scans: Three patterns of enhancement relative to the thyroid gland justify a three-phase protocol. Radiology. 2015; 277(2): 454–62. DOI: 10.1148/radiol.2015142393 26024308

[8] Hoang JK, Sung WK, Bahl M, Phillips CD. How to perform parathyroid 4D CT: Tips and traps for technique and interpretation. Radiology. 2014; 270(1): 15–24. DOI: 10.1148/radiol.13122661 24354373

